# Impact of previous exposure to systemic corticosteroids on unfavorable outcome in patients hospitalized for COVID-19

**DOI:** 10.1186/s40360-021-00480-3

**Published:** 2021-03-11

**Authors:** Margaux Lafaurie, Guillaume Martin-Blondel, Pierre Delobel, Nassim Kamar, Sandrine Charpentier, Agnès Sommet, Guillaume Moulis, and the Covid-Clinic-Toul investigators, Muriel Alvarez, Jacques Amar, Michel Attal, Laurent Balardy, Frédéric Balen, Odile Beyne-Rauzy, Ourdia Bouali, Fanny Bounes, Christophe Bureau, Louis Buscail, Lionel Calviere, François Chollet, Isabelle Claudet, Arnaud Constantin, Alexa Debard, Karen Delavigne, Arnaud Del Bello, Julien Delrieu, Alain Didier, Stanislas Faguer, Marie Faruch, Olivier Fourcade, Bernard Georges, Etienne Grunenwald, Sophie Guyonnet, Hélène Hanaire, Christophe Hein, Jacques Izopet, Olivier Lairez, Pierre Mansat, Vincent Minville, Jérémie Pariente, Carle Paul, Pierre Payoux, Marie-Léa Piel-Julian, Grégory Pugnet, Christian Recher, Agnès Ribes, Yves Rolland, Adeline Ruyssen-Witrand, Jean Sabatier, Laurent Sailler, Jean-Pierre Salles, Nicolas Sans, Marion Secher, Annick Sevely, Stein Silva, Claire Thalamas, Olivier Toulza

**Affiliations:** 1 Service de Pharmacologie Médicale, Centre Hospitalier Universitaire de Toulouse, Faculté de Médecine, 37 allées Jules Guesde, 31000 Toulouse, France; 2 Centre d’investigation clinique 1436, axe pharmacoépidémiologie, Centre Hospitalier Universitaire de Toulouse-Purpan, place du Dr Baylac, TSA40031, 31059 Toulouse, Cedex 9 France; 3 Service des Maladies Infectieuses et Tropicales, Centre Hospitalier Universitaire de Toulouse-Purpan, place du Dr Baylac, TSA40031, 31059 Toulouse, Cedex 9 France; 4 UMR INSERM/CNRS 1043, Centre de Physiopathologie Toulouse Purpan, 330 avenue de Grande-Bretagne, 31059 Toulouse, Cedex 9 France; 5 Service de Néphrologie et Transplantation d’Organes, Centre Hospitalier Universitaire de Toulouse-Rangueil, 1, avenue du Professeur Jean Poulhès - TSA 50032, 31059 Toulouse cedex 9, France; 6 INSERM U1043, IFR –BMT, Université Paul Sabatier, Toulouse, France; 7 Service des Urgences, Centre Hospitalier Universitaire de Toulouse, Toulouse, France; 8 Service de Médecine Interne, salle Le Tallec, Centre Hospitalier Universitaire de Toulouse-Purpan, place du Dr Baylac, TSA40031, 31059 Toulouse, Cedex 9 France

**Keywords:** SARS-COV-2, COVID-19, Systemic corticosteroids, Mortality, Intensive care unit, Pharmacoepidemiology

## Abstract

**Background:**

The impact of prior exposure to systemic corticosteroids on COVID-19 severity in patients hospitalized for a SARS-CoV-2 pneumonia is not known. The present study was designed to answer to this question.

**Methods:**

The population study was the Covid-Clinic-Toul cohort which records data about all hospitalized patients with a positive reverse transcriptase polymerase chain reaction for a SARS-CoV-2 infection at Toulouse University hospital, France. Exposure to systemic corticosteroids was assessed at hospital admission. A propensity score (PS) according to corticosteroid exposure was calculated including comorbidities, clinical, radiological and biological variables that impact COVID-19 severity. The primary outcome was composite, including admission to intensive care unit, need of mechanical ventilation and death occurring during the 14 days after hospital admission. Logistic regression models adjusted for the PS (overlap weighting) provided odds ratios (ORs) and their 95% confidence intervals (95% CIs).

**Results:**

Overall, 253 patients were included in the study. Median age was 64 years, 140 patients (59.6%) were men and 218 (86.2%) had at least one comorbidity. Seventeen patients (6.7%) were exposed to corticosteroids before hospital admission. Chronic inflammatory disease (*n* = 8) was the most frequent indication. One hundred and twenty patients (47.4%) met the composite outcome. In the crude model, the OR of previous exposure to systemic corticosteroids was 1.64; 95% CI: 0.60–4.44. In the adjusted model, it was 1.09 (95% CI: 0.65–1.83).

**Conclusion:**

Overall, this study provide some evidences for an absence of an increased risk of unfavorable outcome with previous exposure to corticosteroids in the general setting of patients hospitalized for COVID-19.

**Supplementary Information:**

The online version contains supplementary material available at 10.1186/s40360-021-00480-3.

## Background

Corticosteroid-based therapy is used to treat patients with severe coronavirus disease 2019 (COVID-19) to reduce inflammatory lung injury, notably when a major cytokine reaction is responsible for clinical worsening [1–4]. A recent prospective meta-analysis of clinical trials conducted by the World Health Organization (WHO) Rapid Evidence Appraisal for COVID-19 Therapies (REACT) Working Group showed that administration of systemic corticosteroids in critically ill patients with COVID-19, compared with usual care or placebo, was associated with lower 28-day all-cause mortality [5]. Moreover, exposure to systemic corticosteroids before COVID-19, responsible for immunosuppression, has been hypothesized to be associated with severe forms of COVID-19. Systemic exposure to corticosteroids has been associated with an increased risk of hospitalization in patients with rheumatic diseases (≥10 mg/day in prednisone-equivalent dosage, OR: 2.05; 95% CI: 1.06–3.96) [6]. A major increased risk of severe COVID-19 (defined by admission in intensive care unit – ICU, need of mechanical ventilation or death) was found in patients with chronic inflammatory bowel disease (OR: 6.9; 95% CI: 2.3–20.5) [7]. These studies were focused on patients with some autoimmune diseases and were not adjusted for clinical, biological and radiological markers of COVID-19 severity. The impact of previous exposure to corticosteroids in the general setting of patients hospitalized for COVID-19 is unknown.

We aimed to assess the impact of prior exposure to systemic corticosteroids on COVID-19 severity in patients hospitalized for reverse transcriptase polymerase chain reaction (RT-PCR)-proven SARS-CoV-2 infection.

## Methods

### Study population

The study was conducted within the Covid-Clinic-Toul cohort which records data about all hospitalized patients with a positive RT-PCR for a SARS-CoV-2 infection at Toulouse University hospital, France [8, 9]. This cohort has been approved by institutional review board (n°RnIPH 2020–31), in accordance with French law. All patients, or their representatives, were informed by a letter given at admission to hospital and/or sent to their place of residency. Exclusion criterion was opposition to data collection. We selected the patients included up to April 20, 2020 and with a chest computed tomography (CT) scan at admission. Patients from April 1st were prospectively included.

### Exposure

Exposure to systemic corticosteroid at hospital admission was assessed by physicians and then extracted from electronic medical records. Drug, dosage, duration of use (categorized as short-term exposure for < 7 days vs ≥ 7 days) and indication were described.

### Outcome

The primary outcome was composite, including admission to ICU, need of mechanical ventilation and death occurring during the 14 days after hospital admission.

### Covariables

The following variables were assessed at hospital admission: age (≥65 years vs. < 65 years), sex, presence of hypertension, cardiovascular disease, cerebrovascular disease, chronic pulmonary disease, chronic liver disease, chronic kidney disease, diabetes, cancer, overweight, immunosuppression (excluding exposure to corticosteroids), oxygen saturation ≤ 92% or need of oxygen therapy, lymphopenia (< 1.5 × 109/L), thrombocytopenia (< 150 × 109/L), C-reactive protein (≥50 mg/L vs. < 50 mg/L), extension of ground glass opacities at chest CT-scan categorized by absence or mild involvement (< 25% of lung parenchyma) vs. moderate to critical involvement (≥25%).

### Statistical analyses

For descriptive analyses, continuous variables were expressed by mean and standard deviation or median and interquartile range (IQR) depending on their distribution, and categorical variables by percentages. For comparatives analyses, multiple imputation (*n* = 5) was used to handle missing values [10]. A propensity score (PS) was calculated based on the covariables listed above [11]. Analyses were conducted using logistic regression models and were adjusted with overlap weighting (OW) on the PS, providing odds ratios (ORs) and their 95% confidence intervals (95% CIs) [12]. OW allows adjustment in population with large differences in covariables, by emphasizing the target population with the most overlap in observed characteristics between exposed and unexposed patients, and down-weighting the tails units. Exposed patients were weighted by the probability of not receiving corticosteroids (1 − PS) and unexposed patients were weighted by the probability of receiving corticosteroids (PS) [13]. Statistical analyses were performed using SAS V9.4™ (SAS institute, Cary, NC, USA).

## Results

### Study population

Overall, 253 patients were included in the study. Characteristics of the patients are presented in Table 1. Median age was 64 years (IQR: 54–76), 140 patients (59.6%) were men, and 218 (86.2%) presented at least one comorbidity. The median duration of symptoms at the time of admission to hospital was 7 days (IQR: 4–10).

**Table 1:** Characteristics of the patients hospitalized for COVID-19 included in the study (*n* = 253)

	Total (n = 253)	Exposure to corticosteroids		Admission to ICU, mechanical ventilation, or death during the first 14 days	
		No (***n*** = 236)	Yes (n = 17)	No (***n*** = 133)	Yes (***n*** = 120)
**Age** (years)					
Median (IQR)	65 (54–76)	64 (54–76)	73 (62–82)	62 (50–75)	68 (58–78)
≥ 65 years, n (%)	128 (50.6)	117 (49.6)	11 (64.7)	59 (44.4)	69 (57.5)
**Sex**					
Male, n (%)	150 (59.3)	141 (59.7)	9 (52.9)	69 (51.9)	81 (67.5)
Female, n (%)	103 (40.7)	95 (40.3)	8 (47.1)	64 (48.1)	39 (32.5)
**Comorbidities**					
≥ 1 comorbidity, n (%)	218 (86.2)	201 (85.2)	17 (100)	111 (83.5)	107 (89.2)
Overweight, (BMI: 25–30 kg/m^2^), n (%)	85 (36.2)	79 (35.6)	6 (46.2)	43 (36.1)	42 (36.2)
Obesity, (BMI ≥ 30 kg/m^2^), n (%)	68 (28.9)	65 (29.3)	3 (23.1)	28 (23.5)	40 (34.5)
Hypertension, n (%)	102 (40.3)	92 (39.0)	10 (58.8)	46 (34.6)	56 (46.7)
Heart failure, n (%)	10 (4.0)	9 (3.8)	1 (5.9)	5 (3.8)	5 (4.2)
History of coronary disease, n (%)	24 (9.5)	20 (8.5)	4 (23.5)	9 (6.8)	15 (12.5)
History of cardiac surgery, n (%)	3 (1.2)	2 (0.9)	1 (5.9)	2 (1.5)	1 (0.8)
History of cerebrovascular disease, n (%)	16 (6.3)	14 (6.0)	2 (11.8)	8 (6.0)	8 (6.7)
Diabetes, n (%)	49 (19.4)	43 (18.2)	6 (35.3)	20 (15.0)	29 (24.2)
Chronic lung disease, n (%)	54 (21.3)	45 (19.7)	9 (52.9)	24 (18.1)	30 (25.0)
Chronic kidney disease, n (%)	23 (9.1)	19 (8.1)	4 (23.5)	11 (8.3)	12 (10.0)
Chronic liver disease, n (%)	2 (0.8)	1 (0.4)	1 (5.9)	0 (0)	2 (1.7)
Malignancy, n (%)	27 (10.7)	22 (9.3)	5 (29.4)	12 (9.0)	15 (12.5)
Immunosuppression, n (%)	20 (7.9)	10 (4.2)	10 (58.8)	8 (6.0)	12 (10.0)
**Time between first symptoms and hospital admission**, median (IQR)^a^	7 (4–10)	7 (5–10)	4 (2–4)	7 (4–10)	7 (4–9)
**At hospital admission**					
Oxygen saturation ≤ 92% or need of oxygen therapy, n (%) ^a^	116 (46.2)	110 (46.8)	6 (37.5)	33 (25.0)	83 (69.8)
C-reactive protein level, > 50 mg/L, n (%) ^a^	129 (51.8)	122 (52.6)	7 (41.2)	53 (39.9)	76 (65.5)
Platelets count, < 150 × 10^9^/L, n (%) ^a^	63 (25.3)	58 (25.0)	5 (29.4)	20 (15.3)	43 (36.4)
Lymphocytes, < 1.5 × 10^9^/L, n (%) ^a^	185 (83.0)	173 (83.2)	12 (80.0)	84 (74.3)	101 (91.8)
**Chest CT-scan severity score**					
Absence or mild, n (%)	44 (17.4)	42 (17.8)	2 (11.8)	27 (20.3)	17 (14.2)
Moderate, severe, or critical, n (%)	209 (82.6)	194 (82.2)	15 (88.2)	106 (79.7)	103 (85.8)
**Exposure to corticosteroids, n (%)**	17 (6.7)	–	17 (100)	7 (5.3)	10 (8.3)
**Composite outcome, n (%)**	120 (47.4)	110 (46.6)	10 (58.8)	–	120 (100)
Admission to ICU, n (%)	109 (43.1)	102 (43.2)	7 (41.2)	–	109 (90.8)
Mechanical ventilation, n (%)	61 (24.1)	58 (24.6)	3 (17.7)	–	61 (50.8)
Death, n (%)	19 (7.5)	14 (5.9)	5 (29.4)	–	19 (15.8)

*Abbreviations*: *BMI* body mass index, *CT* computed tomography, *ICU* intensive care unit, *IQR* interquartile range

^a^Missing data: body mass index, *n* = 18; time between first symptoms and hospital admission, n = 2; oxygen saturation, n = 2; C-reactive protein, n = 2, platelets count, n = 4; lymphocytes, *n* = 30

### Exposure to corticosteroids

Seventeen patients (6.7%) were exposed to corticosteroids before hospital admission. Their characteristics are presented in Table 2. The most frequent corticosteroid was prednisone (*n* = 9). Fifteen patients (83.3%) had an exposure to corticosteroids ≥7 days; indications were chronic inflammatory disease (*n* = 8), solid organ transplantation (*n* = 4) and malignancies (n = 4). As compared with non-exposed patients, those exposed to corticosteroids were older (≥65 years: 64.7% vs 49.6%), with a cause of immunosuppression (58.8% vs 4.2%), chronic lung disease (52.9% vs 19.7%) and hypertension (58.8% vs 39.0%). However, clinical, radiological, and biological markers of COVID-19 severity at hospital admission were comparable between the two groups (Table 1).

**Table 2:** Characteristics of the patients exposed to corticosteroids included in the study

Patient	Age range (years)	Indication	Drug	Daily dose, mg	Duration	Comorbidities	Clinical finding	Biological findings	Chest CT-scan severity score	Outcome
#1	31–40	Multiple myeloma	Dexamethasone	40	3 months	Overweight, cancer, multiple myeloma	–	–	Moderate	No
#2	61–70	Crohn disease	Prednisolone	NA	Long-term	Overweight, chronic lung disease, Crohn disease	Need of oxygen	Lymphopenia, C-reactive protein ≥50 mg/L	Severe	ICU, mechanical ventilation, death
#3	81–90	Horton disease	Prednisolone	2.5	Long-term	Hypertension, coronaropathy, diabetes	SaO2 ≤ 92%	Lymphopenia, thrombocytopenia, C-reactive protein ≥50 mg/L	Severe	Death
#4	71–80	Glioblastoma	Prednisone	20	1 year	Malignancy	–	Lymphopenia	Moderate	Death
#5	51–60	Bronchitis	Prednisolone	60	4 days	Overweight, hypertension	–	–	Moderate	No
#6	81–90	Sarcoidosis	Prednisone	20	4 days	Overweight, hypertension, sarcoidosis, sleep apnea	SaO2 ≤ 92%	C-reactive protein ≥50 mg/L	Moderate	No
#7	61–70	Renal transplant	Prednisone	5	7 years	Overweight, hypertension, sleep apnea, diabetes, chronic kidney disease, renal transplant	–	Lymphopenia, thrombocytopenia	Moderate	ICU
#8	41–50	Cardiac transplant	Prednisone	5	11 years	Cardiac surgery, cardiac transplant	–	–	Absence	No
#9	81–90	Giant cell arteritis	Prednisone	3	4 years	Obesity, hypertension, heart failure, coronaropathy, sleep apnea, chronic kidney disease, diabetes, giant cell arteritis	SaO2 ≤ 92%	Lymphopenia, C-reactive protein ≥50 mg/L	Moderate	ICU, Death
#10	91–100	Lymphoma	Prednisolone	60	8 months	Chronic lung disease, diabetes, lymphoma	–	Lymphopenia	Moderate	No
#11	21–30	Crohn disease	Prednisolone	25	5 months	Immunosuppression	–	C-reactive protein ≥50 mg/L	Moderate	No
#12	71–80	Oesophageal adenocarcinoma	Betametasone	0.4 (30 drops)	3 months	Overweight, coronaropathy, sleep apnea, malignancy	–	Lymphopenia, thrombocytopenia,	Moderate	ICU
#13	71–80	Renal transplant	Prednisone	5	5 months	Hypertension, chronic kidney disease, chronic liver disease, diabetes, renal transplant	–	Lymphopenia, C-reactive protein ≥50 mg/L	Moderate	ICU, mechanical ventilation
#14	61–70	Renal transplant	Prednisone	5	2 years	Hypertension, cerebrovascular disease, sleep apnea, chronic kidney disease, renal transplant	–	Lymphopenia, thrombocytopenia,	Moderate	ICU, mechanical ventilation
#15	71–80	Rheumatoid arthritis	NA	NA	6 weeks	Hypertension, rheumatoid arthritis	–	Lymphopenia	Moderate	No
#16	61–70	Sarcoidosis	Prednisone	6.5	1 year	Obesity, hypertension, COPD, sarcoidosis	–	Lymphopenia	Mild	ICU, death
#17	81–90	Dermatomyositis	Prednisone	70	1 month	Hypertension, lung fibrosis, malignancy dermatomyositis	Need of oxygen	Lymphopenia, thrombocytopenia, C-reactive protein ≥50 mg/L	Severe	Death

*Abbreviation*: *COPD* chronic obstructive pulmonary disease, *NA* Not available

### Outcome

One hundred and twenty patients (47.4%) met the composite outcome during the first 14 days of hospitalization; 61 (24.1%) required mechanical ventilation and 19 (7.5%) died (Table 1). The median time between admission and outcome occurrence was 1 day (IQR: 0–3 days). Ten patients exposed to corticosteroids (58.8%), all with an exposure ≥7 days, met the composite outcome (Table 2).

### Comparative analyses

In the crude model, the OR of exposure to systemic corticosteroid at the time of admission to hospital with outcome occurrence was 1.64; 95% CI: 0.60–4.44. The PS distribution is presented in Supplementary Fig. S1. The PS was efficient in establishing balance for each covariable (data not shown). In the adjusted model with OW on the PS, the OR was 1.09 (95% CI: 0.65–1.83).

## Discussion

In this study, we found a trend for an increased risk of poor outcome in COVID-19 hospitalized patients in case of previous exposure to corticosteroid before hospital admission. However, after adjustment for potential confounders, we found no evidence for an increased risk. This result differs from the two previously quoted studies in patients with rheumatic disease or chronic inflammatory bowel disease [6, 7]. However, these were other settings, with no adjustment for other COVID-19 severity markers at the time of admission. Moreover, in the study conducted in patients with chronic inflammatory bowel disease, comorbidities were included quantitatively [7]. However, some comorbidities are expected to be more related with both exposure to corticosteroids and disease severity. That’s why we included each comorbidity in the PS calculation. Of note, patients exposed to corticosteroids in our cohort had more frequently chronic lung disease, hypertension, and cause of immunosuppression only.

This study conducted in the Covid-Clinic-Toul cohort presented strengths. The cohort is a clinical cohort with most of the data prospectively collected. Exposure to systemic corticosteroids was exhaustively assessed for each patient. Missing values were very rare and handled by multiple imputation. Adjustment using OW on the PS provided risk estimation by minimizing confusion bias due to important differences in the characteristics between exposed and unexposed patients.

Our study had several limitations. Data were restricted to a single hospital center. Only 6.7% (*n* = 17) of the patients were exposed to corticosteroids before hospital admission. This low sample size limited the interpretation of the results. Therefore, we could have only detected a major effect of corticosteroids on unfavorable outcome. Subgroup analyses by corticosteroids dosage, duration of exposure, and indications cannot be conducted due to this low number of exposed patients. Of note, 8 of the exposed patients (47%) in our cohort had a daily prednisone equivalent dosage < 10 mg, which was not associated with an increased rate of hospitalization in the study in patients with rheumatic disease [6]. Because previous exposure to systemic corticosteroids is strongly associated with their indication and because subgroups analyses were not possible, results need to be interpreted cautiously. Finally, we cannot exclude the presence of unmeasured confounding factors like smoking. However, these factors are certainly related to comorbidities included in the PS and it is not clear whether they impact COVID-19 severity.

## Conclusion

Overall, this study provide some evidences for an absence of an increased risk of unfavorable outcome with previous exposure to corticosteroids in the general setting of patients hospitalized for COVID-19.

## Supplementary Information

**Additional file 1: Figure S1.** Propensity score distribution according to exposure to systemic corticosteroids prior to hospitalization by each imputation of missing data (5 imputations, panels A to E) in the study population (n=253). Blue bars: patients unexposed to systemic corticosteroids. Red bars: patients exposed to systemic corticosteroids.

## Data Availability

The data that support the findings of this study are available on request from the corresponding author. The data are not publicly available due to privacy and ethical restrictions. The data management and statistical analysis code is available on request from the corresponding author.

## Abbreviations

CT: Computed tomography
ICU: Intensive care unit
OW: Overlap weighting
PS: Propensity score
RT-PCR: reverse transcriptase polymerase chain reaction
