# Supplementary material for: Impact of previous exposure to systemic corticosteroids on unfavorable outcome in patients hospitalized for COVID-19
Source: BMC Pharmacol Toxicol. 2021 Mar 11;22:14. doi: 10.1186/s40360-021-00480-3 (PMC7948656; doi:10.1186/s40360-021-00480-3)
Supplement: Supplementary file 1 — Additional file 1: Figure S1. Propensity score distribution according to exposure to systemic corticosteroids prior to hospitalization by each imputation of missing data (5 imputations, panels A to E) in the study population (n=253). Blue bars: patients unexposed to systemic corticosteroids. Red bars: patients exposed to systemic corticosteroids. [file 40360_2021_480_MOESM1_ESM.docx]

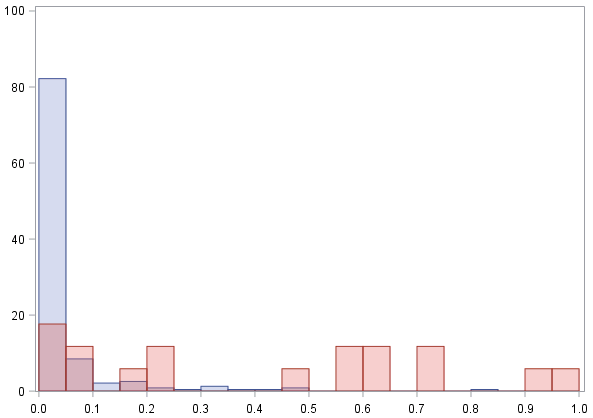

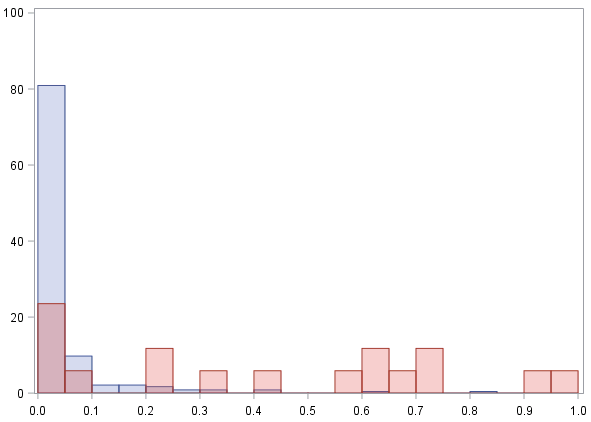

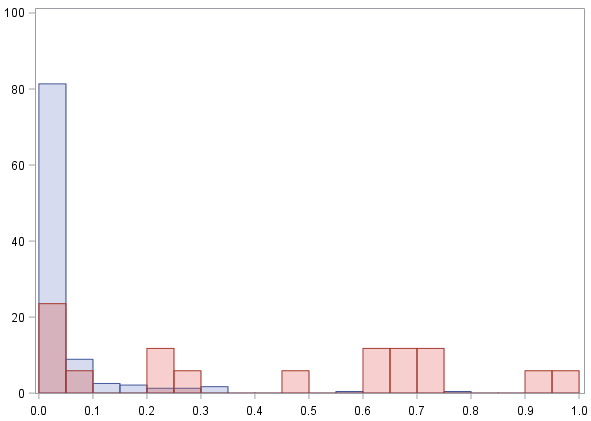

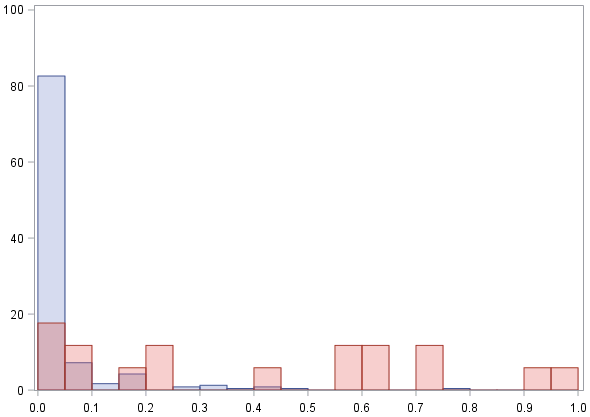

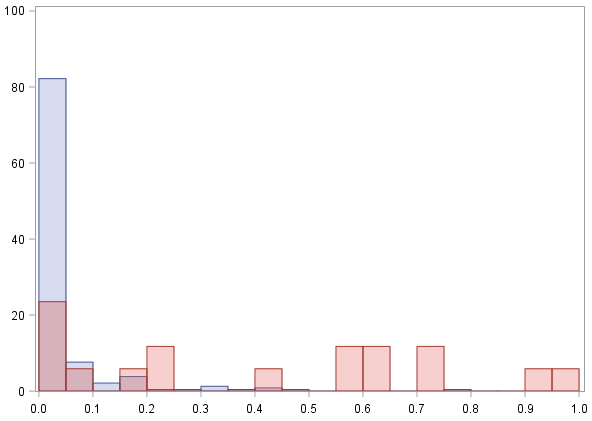


Percentage of patients

Propensity score

**A**

Percentage of patients

Propensity score

**B**

**C**

Percentage of patients

Propensity score

Percentage of patients

**D**

Propensity score

**E**

Percentage of patients

Propensity score

**Figure S1. Propensity score distribution according to exposure to systemic corticosteroids prior to hospitalization by each imputation of missing data (5 imputations, panels A to E) in the study population (n=253). Blue bars: patients unexposed to systemic corticosteroids. Red bars: patients exposed to systemic corticosteroids.**
